# A Partially Hydrolyzed Whey Infant Formula Supports Appropriate Growth: A Randomized Controlled Non-Inferiority Trial

**DOI:** 10.3390/nu12103056

**Published:** 2020-10-06

**Authors:** Eva Karaglani, Inge Thijs-Verhoeven, Marjan Gros, Christina Chairistanidou, Giorgos Zervas, Christina Filoilia, Tarek-Michail Kampani, Vasileios Miligkos, Maria Matiatou, Stavroula Valaveri, Alexandros Sakellariou, Georgios Babilis, Rolf Bos, Yannis Manios

**Affiliations:** 1 Department of Nutrition and Dietetics, School of Health Science and Education, Harokopio University, 70 El. Venizelou Ave, 17671 Athens, Greece; ekaragl@hua.gr (E.K.); christina.chairistanidou@gmail.com (C.C.); chfiloilia@gmail.com (C.F.); moxaxmet@otenet.gr (T.-M.K.); pediatrics.sakellariou@gmail.com (A.S.); 2 FrieslandCampina, Stationsplein 1, 3818 LE Amersfoort, The Netherlands; Inge.Thijs-Verhoeven@frieslandcampina.com (I.T.-V.); Marjan.Gros@FrieslandCampina.com (M.G.); Rolf.Bos@frieslandcampina.com (R.B.); 3 Mitera Hospital, 6 Erythrou Stavrou Str., 151 23 Athens, Greece; giorgoszervas@gmail.com (G.Z.); miligkosb@gmail.com (V.M.); dr.matiatou@gmail.com (M.M.); svalaveri@yahoo.gr (S.V.); gbampilis@yahoo.gr (G.B.)

**Keywords:** infant formula, protein hydrolysate, growth, partially hydrolyzed formula, anthropometry

## Abstract

The aim of the current study was to investigate the effects of a partially hydrolyzed whey infant formula (PHF) on growth in healthy term infants as compared to a standard infant formula with intact protein (IPF). In a double-blind, non-inferiority, randomized controlled trial, a total of 163 healthy formula-fed infants, 55–80 days old, were recruited and randomly allocated to either the PHF (test) or the IPF (control) group. They were followed up for three months during which they were evaluated monthly on growth and development. In total, 21 infants discontinued the study, while 142 infants completed the study (test *n* = 72, control *n* = 70). The primary outcome was daily weight gain during the three months. Secondary outcomes included additional anthropometric indices at every timepoint over the intervention period. Daily weight gain during the three-month intervention period was similar in both groups with the lower bound of 95% confidence interval (CI) above the non-inferiority margin of −3 g/day [mean difference (95% CI) test vs. control: −0.474 (−2.460, 1.512) g/day]. Regarding secondary outcomes, i.e., infants’ weight, length, head circumference, body mass index (BMI), and their Z-scores, no differences were observed between the two groups at any time point. The PHF resulted in similar infant growth outcomes as the standard IPF. Based on these results, it can be concluded that the partially hydrolyzed whey infant formula supports adequate growth in healthy term infants.

## 1. Introduction

Optimal feeding practices during early life are of utmost importance to support healthy growth and development in infants [1]. Human milk represents the optimum nutrition throughout infancy and is associated with several short- and long-term benefits for both the child and the mother [1,2,3]. However, when breastfeeding is not feasible, infant formulas (IF) are the best alternative.

Research has shown that infants who are formula-fed weigh more and have a higher risk of obesity later in life compared to breast-fed infants [4,5]. Therefore, protein sources and IF processing technologies have been modified over the past years to optimize both the quality and the quantity of proteins in IF to better suit the nutritional requirements of infants and support more optimal growth. Protein hydrolysis, i.e., where proteins are digested into smaller fragments, peptides, or amino acids, is a frequent modification in IF, particularly those designed for special medical purposes [6]. Depending on the level of hydrolysis, hydrolysates can be classified as partially or extensively hydrolyzed proteins.

Hydrolysate-based formulas have been mainly developed for cow’s milk protein allergy (CMPA) management, as IF containing extensively or partially hydrolyzed proteins are suggested to reduce the risk of developing allergic manifestations during the first four to six months of life [7,8], whilst extensively hydrolyzed formulas are successfully used in symptoms’ management of existing CMPA [9,10]. Furthermore, hydrolysate-based formulas are widely used for preterm infants, when breastfeeding is not available [11,12,13], while some studies suggest potential benefits of partially hydrolyzed formulas (PHF) in the dietary management of common functional gastrointestinal symptoms such as fussiness, reflux, and colicky symptoms in formula-fed infants [14,15].

Despite the potential benefits of hydrolyzed protein formulas on CMPA prevention or gastrointestinal tolerance, it still needs to be evaluated whether growth indices remain comparable between infants fed standard intact protein formulas (IPF) and infants fed protein hydrolysate-based IF. For this reason, new European Commission regulations [16], applying to hydrolysate-based formulas from 2021 onwards, require that the safety and suitability of each specific hydrolysate-based IF is evaluated by clinical studies.

The primary objective of the current study was to evaluate the weight gain of healthy term infants consuming a whey-based PHF compared to a standard IPF over a period of three months. The secondary objective included evaluation of additional anthropometric indices at every timepoint over the period of three months.

## 2. Materials and Methods

### 2.1. Study Design and Population

This study was a double-blind, randomized controlled trial with two study arms: The test group consuming the PHF and the control group consuming the IPF. The study was conducted in healthy, full-term, exclusively formula-fed infants. Sampling and recruitment were performed by pediatricians in two cities (Athens and Larissa) in Greece between October 2018 (first subject in) and June 2019 (last subject in), while the overall study period ended in September 2019 (last subject out). Infants were enrolled between the 55th and 80th day of age during routine visits to the pediatricians. The inclusion criteria can be found in Supplementary Methods S1. Written informed consent was obtained from the parent/legal guardian of each infant before any study procedures were initiated.

The study protocol, information letter to the parents/legal guardians, and written informed consent form were approved by Harokopio University’s Ethics Committee (approval code: 62/03-07-2018). The study was conducted in accordance with the guidelines of the Declaration of Helsinki and the International Conference on Harmonization (ICH) guidelines on Good Clinical Practice (GCP) and was registered in the Netherlands Trial Registry (identifier: NL7378 (NTR7586)).

### 2.2. Study Procedures and Formulas

Upon inclusion in the study, subjects were randomized to one of four coded products representing the two study formulas. Randomization was performed centrally, at Harokopio University, by a designated and trained research assistant based on computer-generated schemes. For each pediatrician a distinct randomization table was created to ensure that infants recruited within one site would be appropriately randomized across treatments. Each time a pediatrician recruited an infant, the research assistant at Harokopio University was notified and she randomized the infant into one of the study groups. Next, she informed the pediatrician which coded formula the infant would be provided with, while also arranging delivery of the appropriate formula to the infant’s house.

Formulas were provided for free to the participating families during the three-month study period and were used as the sole source of nutrition for the participating infants. Formula consumption was ad libitum but a feeding table in the “Parent Information Brochure” supported a correct consumption of the study products.

The nutritional compositions of the IF used in this study are compliant to Commission Directive 2006/41/EC of 7 July 2006 amending Council Directive 91/414/EEC to include clothianidin and pethoxamid as active substances and are similar with regards to macro-nutrients, apart from the protein fraction (Table 1; for analytical composition of the two formulas see Table S2). Both IF were cow’s milk based and were produced in the Netherlands by FrieslandCampina and packed in blank tins of 400 g each with a specific identification code at the bottom. All powder properties were identical between the test and control formulas. Parents/legal guardians, investigators, and study support staff were blinded to the formulas. Data analyses were performed with the study groups coded and the code was not broken until the database was locked.

Once the informed consent form was obtained, baseline anthropometric measurements (weight, length, and head circumference) were performed by the pediatrician, while family demographic information, perinatal, and birth characteristics of study participants were also collected. Three follow-up visits were performed thereafter, at the following time-points: Baseline +30, +60, and +90 days, with an allowed deviation of +/−2 days. Formula intake was assessed using a paper diary, which was completed by the parent/legal guardian on seven consecutive days before the visit to the pediatrician. At each visit, the formula intake diary was collected and a clinical examination to obtain anthropometric measurements was performed by the pediatrician. Adverse events (AEs), serious adverse events (SAEs), and medication use were recorded during the follow-up visits and monitored by an independent pediatrician. No code-break requests occurred for AEs or SAEs throughout the study.

### 2.3. Primary and Secondary Outcome Measures

The primary outcome was weight gain (g/day) calculated as the difference in infant weight between the baseline and the 3rd follow-up visit, divided by the number of days between these visits. Secondary outcomes included other anthropometric indices assessed at each follow-up visit: Weight (g), length (cm), head circumference (cm), body mass index (BMI) (kg/m^2^), and their Z-scores (based on the World Health Organization (WHO) child growth standards [17]). More details on the primary and secondary outcome measures can be found in Supplementary Methods S3.

### 2.4. Sample Size and Statistical Analysis

The sample size was determined according to guidelines from the American Academy of Pediatrics Task Force on Clinical Testing of Infant Formulas [18] and as described previously by Puccio et al. (2017) [19]. Specifically, the sample size calculation was based on a non-inferiority test, using a one-sided, two sample *t*-test for the comparison of weight gain at three months of intervention between treatment groups. The PASS (version 15.0.4) software was used. For the margin of non-inferiority, a weight gain of −3 g/day was determined [18]. Assuming a 2.5% significance level, a power of 80% and a standard deviation of 6.1 g/day [19], 66 infants were needed in each formula group. The expected dropout rate was estimated to be 30%, mainly because of non-compliance to the required feeding strategy, thus enrolment of 95 infants per group was planned.

The null hypothesis was that the difference in weight gain between the test and control group would be higher than −3 g/day. The alternative hypothesis of non-inferiority was that the difference in weight gain between the two groups (test minus control) would be smaller than −3 g/day.

For analysis of the primary endpoint, a one-sided statistical significance level of α = 0.025 was used, while for the secondary endpoints, a two-sided statistical significance level of α = 0.05 was used. No correction for multiplicity was done, because there was only one primary parameter and missing data were not imputed.

The primary endpoint (weight gain during the three-month intervention in g/day) was analyzed using an analysis of covariance (ANCOVA) model, with the study formula as a fixed factor and adjustments for multiple covariates, including baseline weight, sex, antibiotic use, birth weight, maternal pre-pregnancy BMI, father’s current BMI, and average formula intake. The adjusted mean and standard error (SE) of weight gain is reported. The primary endpoint analyses were carried out in both the intention-to-treat (ITT) and per-protocol (PP) analysis sets.

The secondary endpoint analyses were also carried out in both the ITT and the PP analysis sets and were analyzed using a mixed models repeated measures (MMRM) analysis, with the study formula and visit as fixed factors, adjusting for several covariates (see primary outcome) and their interactions.

Data were analyzed independently by the statistical company OCS Life Sciences. The statistical analyses were performed using the SAS software version 9.4 or higher (SAS Institute, Cary, NC, USA).

## 3. Results

### 3.1. Study Population

**Figure 1:**
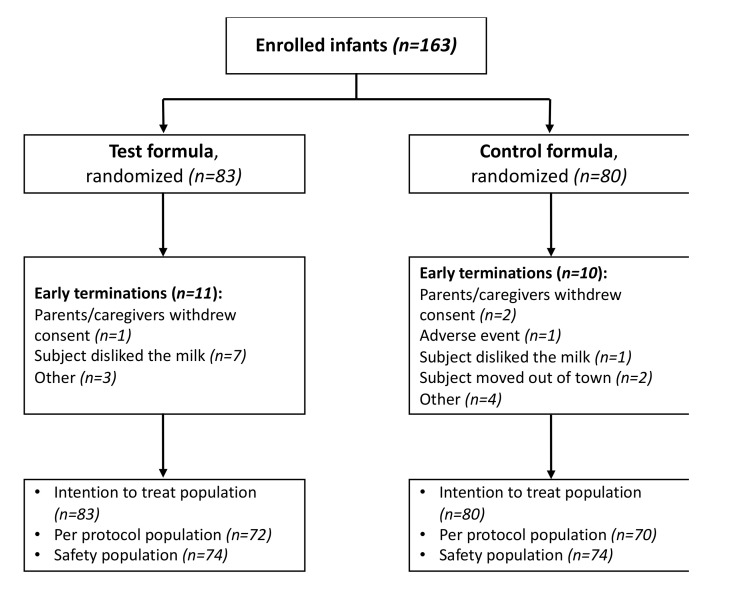
Study flowchart and subjects’ disposition. Test formula: Partially hydrolyzed whey infant formula; control formula: Standard intact protein formula.

A total of 163 infants were enrolled and randomized into the trial (83 test formula, 80 control formula; Figure 1). Considering that the dropout rate was much lower than 30%, the minimum number of completed subjects needed to reach statistical power (*n* = 66 per treatment group) was achieved earlier than anticipated; therefore, the recruitment was ended before 95 infants were enrolled per treatment group. Of the 163 infants recruited, 142 infants completed the study (72 test formula, 70 control formula), while 21 infants (11 test formula, 10 control formula) discontinued the study. The reasons for discontinuation for each study group can be seen in Figure 1.

Demographic, perinatal, and birth characteristics were comparable between the groups, except for years of maternal education (Table 2). Baseline characteristics also did not differ between the groups except for weight at baseline, indicating that infants in the control group had a higher weight at baseline than infants in the test group (Table 2).

### 3.2. Weight Gain and Growth

In the PP population, the adjusted mean (SE) weight gain during the three-month intervention period was 24.06 (2.64) g/day for infants fed the test formula and 24.54 (2.51) g/day for those fed the control (Table 3). The mean difference (95% CI) in weight gain between groups was −0.474 (−2.460, 1.512) g/day, with the lower limit of the 95% CI above the predefined non-inferiority margin of −3 g/day, rejecting the null hypothesis and indicating a similar weight gain in the two groups. Results were similar in the ITT population.

Regarding the secondary outcomes, in the PP population, there were no significant differences between the two groups at any follow-up visit in weight, length, head circumference, and BMI (Table S4). Furthermore, no treatment effect over time was observed for any of those indices during the three-month intervention period (Table S4). Similar results were obtained in the ITT population (Table S5). Regarding gains in weight (in g/day) from baseline to the 1st or 2nd follow-up visits, no differences were observed between the two groups (Table S6). Likewise, no differences were found for gains in length (in cm/day) between the two groups over the three-month period (from baseline to each of the three monthly follow-up assessments; Table S6). Gains in head circumference (in cm/day) were slightly lower in the test group compared to the control from baseline to the 1st follow-up visit, but no differences were observed between the two groups thereafter (from baseline to the 2nd and 3rd follow-up assessments; Table S6). All the above findings were consistent between the PP and ITT populations.

Similarly, mean weight-for-age, length-for-age, head circumference-for-age, and BMI-for-age Z-scores did not differ between the two groups at any follow-up visit. Only weight-for-length Z-scores were slightly lower in the test group compared to the control at the 1st follow-up visit, but no differences were observed between the two groups thereafter. Results were again similar in the ITT population. Figure S7 presents the relevant Z-scores of both groups during the study period in comparison with the WHO growth standards for female and male infants based on the crude (unadjusted) data. All Z-scores were tracked closely with the WHO growth standards [20].

### 3.3. Formula Intake and Safety Parameters

Infants in the control group had a higher weekly formula consumption (≈ +10.5%) compared to infants in the test group at all three follow-up measurements (Table 4). However, when the daily formula intake was corrected for body weight, no differences were observed between the two groups at all time points (Table 4).

Overall, 16 AEs occurred in the total study cohort, half of which (*n* = 8) occurred in the test formula group and half of which (*n* = 8) occurred in the control formula group. All the AEs and SAEs were unrelated to the intervention indicating no formula related risk (Table S8).

## 4. Discussion

The present study demonstrated a non-inferior weight gain between infants consuming a whey-based PHF and infants consuming a standard IPF during the three-month trial duration. Moreover, no differences were observed between the two groups on any growth measurements (weight, length, head circumference, and BMI), while overall growth trajectories were within the normal range based on WHO growth standards [20]. The two formulas used in the current study were similar with regards to macro-nutrients, apart from the protein fraction, and were therefore isocaloric, providing 66 kcal per 100 mL. The slight differences in galacto-oligosaccharides, which are non-digestible oligosaccharides, and some micro-nutrients could not have affected the weight gain of infants. Therefore, as hypothesized, the absence of differences on growth outcomes between the two formula groups suggests that substituting intact protein with partially hydrolyzed protein in IF is safe and supports appropriate growth in healthy infants.

Regarding the primary outcome, the current results are consistent with previous studies. In the study by Wu et al. (2018) [21], no differences were observed in daily weight gain in healthy term infants fed a PHF compared to infants fed an IPF or breast milk from enrolment to the 7th and 13th week of age. Florendo et al. (2009) [22] compared the effects of a standard non-hydrolyzed whey–casein formula to a preterm PHF for three weeks. No differences in daily weight gain were observed between the two groups during the 3-week study duration. In the German Infant Nutritional Intervention Study (GINI) [23], four different types of formulas were assessed, as well as a breast milk reference group; these formulas were either a whey PHF, an extensively hydrolyzed whey formula, an extensively hydrolyzed casein formula, or a regular IPF. Weight gain during the first four and six months of life showed no differences in infants with atopic heredity who consumed either breast milk or one of the formula groups, except for the extensively hydrolyzed casein formula which showed a transient lower weight gain. Despite the diverse study designs and IF used, it has been shown overall that no differences in weight gain were observed when healthy infants were fed either PHF or regular IPF during early infancy.

The findings of the current study on secondary outcomes, i.e., weight, length, head circumference, and BMI showed no differences between the test and control groups at all three time points. These findings are also in line with the results reported for those indices by Wu et al. (2018) [21], Florendo et al. (2009) [22], and the GINI study [23] described above. Similar findings were also reported in other studies [24,25]. Although difficult to directly compare due to methodological variations, previous studies and current results collectively suggest that weight, length, head circumference, and BMI of infants fed either protein hydrolysate-based formulas or regular IPF do not show any differences during the first months of life.

Regarding mean Z-scores (weight-for-age, length-for-age, head circumference-for-age, weight-for-length, and BMI-for-age), the current study found no differences between the two study groups during the three-month period. Furthermore, all mean Z-scores were within the normal range based on WHO growth standards [20]. Again, consistent results have been reported by previous studies as mentioned above [21,22,23,24,25]. However, in the study by Menella et al. [26], Z-scores trajectories across infants aged 2.5 to 7.5 months showed significantly higher weight-for-age Z-scores in the infants fed a regular IPF compared to infants fed a PHF. Weight gain was accelerated in the former, whereas it was normative in the latter. Still, the differences observed in weight gain rates in this study could be attributed to the difference in the amount of formula consumed between the two study groups, since infants in the protein hydrolysate group consumed less formula to satiation than did regular formula-fed infants across the study period [26].

Regarding formula intake, a significant group effect was observed in the present study, with infants in the test group consuming less formula than infants in the control group at each monthly follow-up assessment. This phenomenon, also observed in the study by Menella et al. [26], could be attributed to the sensory characteristics of the two formulas, as infants may dislike the taste of protein hydrolysates, occurring due to the increased levels of free amino acids and small peptides with a bitter taste, and consequently consume less. This is further supported by the fact that the main reason for dropping out of the study in the test group was that infants disliked the test formula. Still, the overall drop-out rate was much lower than anticipated. Furthermore, it has been shown that the sooner a hydrolysate-based formula is introduced in an infant’s diet, the more accepted it is by the infant [27]. Therefore, considering that infants in the present study had a mean age of 67 days at baseline, the test formula might have not been equally accepted by the infants as the control formula. Another potential explanation could be that hydrolyzed proteins have been shown to promote satiation signals and stimulate earlier meal termination in infants who consume protein hydrolysate-based formulas [28,29]. Nevertheless, the lower formula intake observed in infants consuming the test formula did not affect weight gain or other growth outcomes at any time point compared to the control formula in the current study, and supported normative growth based on WHO growth standards [20].

Among the strengths of the current study are the double-blind study design and the standardized procedure followed for data collection. Specifically, recruitment was performed by several pediatricians, but infants’ growth was prospectively assessed by the same pediatrician who enrolled them in the study, during the entire study period. Still, the large number of pediatricians involved in the study could introduce some variation in the measurements performed. To ensure comparability of the anthropometric data obtained among sites, all pediatricians were trained to follow the same standardized procedures for anthropometrics, while intra- and inter-observer reliability was also periodically assessed. Another strength of the present study was that, as described in the methods section, different randomization tables were created for each pediatrician to ensure that infants would be appropriately randomized across treatments within each site.

## 5. Conclusions

The current study demonstrated that weight gain, as well as other growth outcomes did not differ between infants consuming the whey-based PHF and those consuming the IPF. All the Z-score indices obtained were within the normal range of WHO growth standards. Based on these results, it can be concluded that the IF with partially hydrolyzed protein supports appropriate growth in healthy term infants.

## Figures and Tables

**Table 1:** Composition of the study formulas (per 100 mL).

	Test Formula	Control Formula
Energy (kcal)	66	66
Intact protein (g)		1.4
Casein		0.57
Whey		0.85
Whey protein hydrolysate (g)	1.6	
Fat (g)	3.5	3.5
DHA (mg)	6.9	6.9
AA (mg)	6.9	6.9
Carbohydrates GOS (g)	7.0 0.2	7.0 0.4
Ca (mg)	50	56
P (mg)	30	31
Na (mg)	20	23
Fe (mg)	0.78	0.77
Vitamin D (μg)	1.2	1.1

Test formula: Partially hydrolyzed whey infant formula; control formula: Intact protein formula; AA: Arachidonic acid; DHA: Docosahexaenoic acid; GOS: Galacto-oligosaccharides; Ca: Calcium; P: Phosphorus; Na: Sodium; Fe: Iron.

**Table 2:** Demographic, perinatal, and baseline characteristics of study infants.

	Group	
	Test (*N* = 83)	Control (*N* = 80)
Infant characteristics		
Age at baseline (days), mean (SD)	66.9 (7.5)	67.1 (7.5)
Gender (female), *n* (%)	41 (49.4)	39 (48.8)
Weight at baseline (g), mean (SD)	5223 (694) ^1^	5443 (639)
Length at baseline (cm), mean (SD)	59.12 (2.34)	59.26 (2.94)
Head Circumference at baseline (cm), mean (SD)	38.90 (1.31)	38.74 (1.23)
Birth weight (g), mean (SD)	3206 (398)	3159 (392)
Gestational age (weeks), mean (SD)	38.3 (1.1)	38.3 (1.1)
Caesarean delivery, *n* (%)	55 (66.3)	52 (65.0)
**Maternal characteristics**		
Age at baseline (years), mean (SD)	32.9 (6.4)	32.7 (5.8)
Parity (primiparous), *n* (%)	41 (49.4)	34 (42.5)
BMI at baseline (kg/m^2^), mean (SD)	26.03 (4.74)	27.07 (5.07)
Education, *n* (%)		
≤12 years	28 (33.7) ^1^	29 (36.2)
13–16 years	53 (63.9) ^1^	40 (50.0)
>16 years	2 (2.4) ^1^	11 (13.8)
Smoking during pregnancy, *n* (%)	22 (26.5)	16 (20.0)
Single pregnancy, *n* (%)	75 (90.4)	72 (90.0)

^1^*p* < 0.05. Test: Partially hydrolyzed whey infant formula; control: Intact protein formula; N: Number of subjects in analysis population; SD: Standard deviation; BMI: Body mass index.

**Table 3:** Weight gain of study infants from baseline to the 3rd follow-up.

Population	Group	Weight Gain (g/d) Baseline—3rd Follow-Up	Difference between Groups (Test vs. Control)		*p*-Value
		LS mean (SE)	Estimate	95% CI	
PP	Test (*n* = 72)	24.06 (2.635)	−0.474	−2.460, 1.512	0.637
	Control (*n* = 70)	24.54 (2.513)			
ITT	Test (*n* = 83)	23.91 (2.789)	−0.641	−2.480, 1.399	0.535
	Control (*n* = 80)	24.55 (2.659)			

Test: Partially hydrolyzed whey infant formula; control: Intact protein formula; PP: Per protocol; ITT: Intention to treat; CI: Confidence interval; LS mean: Least squares mean; SE: Standard error.

**Table 4:** Formula intake at each follow-up visit by study group.

Daily Formula Intake by Body Weight (mL/g/d)						
	PP Population			ITT Population		
Study Visit	Test	Control		Test	Control	
	LS Mean (95% CI)	LS Mean (95% CI)	*p*-Value	LS Mean (95% CI)	LS Mean (95% CI)	*p*-Value
Follow-up 1	1.00 (0.96, 1.04)	1.02 (0.97, 1.06)	0.651	1.00 (0.96, 1.05)	1.01 (0.97, 1.05)	0.807
Follow-up 2	0.95 (0.90, 0.99)	0.98 (0.94, 1.02)	0.268	0.95 (0.90, 0.99)	0.98 (0.94, 1.03)	0.239
Follow-up 3	0.92 (0.89, 0.96)	0.93 (0.89, 0.97)	0.808	0.92 (0.89, 0.96)	0.93 (0.89, 0.97)	0.808
**Weekly formula intake (mL)**						
	**PP population**			**ITT population**		
	**Test**	**Control**		**Test**	**Control**	
	**Median**	**Median**	***p*-value**	**Median**	**Median**	***p*-value**
Follow-up 1	5757.5	6492.5	<0.001	5797.5	6455.0	0.001
Follow-up 2	6107.5	6880.0	<0.001	6107.5	6860.0	<.001
Follow-up 3	6420.0	7040.0	0.002	6420.0	7040.0	0.002

Test: Partially hydrolyzed whey infant formula; control: Intact protein formula; PP: Per protocol; ITT: Intention to treat; SE: Standard error.

## Supplementary Materials

The following are available online at https://www.mdpi.com/2072-6643/12/10/3056/s1. Methods S1: Inclusion and exclusion criteria; Table S2: Analytical composition of the study formulas (per 100 mL); Methods S3: Primary and secondary outcome measures and statistical analysis; Table S4: Weight, length, head circumference, and BMI at each follow-up visit by study group in the PP population; Table S5: Weight, length, head circumference, and BMI at each follow-up visit by study group in the ITT population; Table S6: Gains in weight, length, and head circumference at each follow-up visit by study group; Figure S7: Anthropometric measurements expressed as Z-scores in comparison with the World Health Organization growth standards; Table S8: Overview of adverse events and serious adverse events that occurred during the trial.
