# Supplementary material for: A Partially Hydrolyzed Whey Infant Formula Supports Appropriate Growth: A Randomized Controlled Non-Inferiority Trial
Source: Nutrients. 2020 Oct 6;12(10):3056. doi: 10.3390/nu12103056 (PMC7650565; doi:10.3390/nu12103056)
Supplement: Supplementary file 1 [file nutrients-12-03056-s001.zip › Methods, Supplementary File 1.docx]

eMethods 1. Inclusion and exclusion criteria

| **Inclusion criteria:**   - Full-term, healthy infants (born at gestational age ≥37 weeks) in the general population - Appropriate for gestational age birthweight (i.e. 10th centile ≤ Birth weight ≤ 90th centile) - Boys and girls - Age at enrolment (baseline measurement): between 55 and 80 days of age - Exclusively formula fed two weeks before inclusion - Exclusively formula fed during the entire intervention period - Parents agreeing to initiate complementary feeding after finalization of the study (endpoint measurements at ~5.5 months of age) - Being available for follow up until the age of approximately 5.5 months - Written informed consent   **Exclusion criteria:**   - Severe acquired or congenital diseases, mental or physical disorders including cow’s milk protein allergy (CMA), lactose intolerance and diagnosed medical conditions that are known to affect growth [i.e. gastrointestinal (GI) disorders] - Illness at screening/inclusion - Incapability of parents to comply with the study protocol - Participation in another clinical trial - Unwillingness to accept the formula supplied by the study as the only formula for their child during study participation |
| --- |
