# Supplementary material for: A Partially Hydrolyzed Whey Infant Formula Supports Appropriate Growth: A Randomized Controlled Non-Inferiority Trial
Source: Nutrients. 2020 Oct 6;12(10):3056. doi: 10.3390/nu12103056 (PMC7650565; doi:10.3390/nu12103056)
Supplement: Supplementary file 1 [file nutrients-12-03056-s001.zip › Table, Supplementary File 2_new.docx]

*eTable 2. Analytical composition of the study formulas (per 100 ml)*

|  | **Test formula** | **Control formula** |
| --- | --- | --- |
| Energy (kcal) | 66 | 66 |
| Intact protein (g) |  | 1.4 |
| Casein |  | 0.57 |
| Whey |  | 0.85 |
| Whey protein hydrolysate (g) | 1.6 |  |
| Fat (g)  DHA (mg)  Arachidonic Acid (mg) | 3.5  6.9  6.9 | 3.5  6.9  6.9 |
| Carbohydrates  GOS (g) | 7.0  0.2 | 7.0  0.4 |
| Calcium (mg) | 50 | 56 |
| Phosphorus (mg) | 30 | 31 |
| Sodium (mg) | 20 | 23 |
| Iron (mg) | 0.78 | 0.77 |
| Copper (μg) | 50 | 47 |
| Potassium (mg) | 65 | 79 |
| Magnesium (mg) | 6 | 6.4 |
| Manganese (μg) | 17 | 16 |
| Zinc (mg) | 0.60 | 0.60 |
| Chlorine (mg) | 42 | 47 |
| Iodine (μg) | 10 | 9 |
| Selenium (μg) | 1.7 | 2.5 |
| Vitamin A (μg-RE) | 70 | 74 |
| Vitamin D (μg) | 1.2 | 1.1 |
| Vitamin E (mg) | 1.3 | 1.7 |
| Vitamin K (μg) | 5.1 | 6.2 |
| Vitamin B1 (μg) | 59 | 57 |
| Vitamin B2 (μg) | 91 | 78 |
| Niacin mg | 0.47 | 0.49 |
| Vitamin B6 (μg) | 39 | 58 |
| Vitamin B12 (μg) | 0.16 | 0.16 |
| Folic acid (μg) | 10 | 11 |
| Pantothenic acid (μg) | 0.33 | 0.40 |
| Biotin (μg) | 1.4 | 1.7 |
| Vitamin C (mg) | 9.1 | 11 |
| Nucleotides (mg) | 3.25 | 3.25 |
| Taurine (mg) | 6 | 7.3 |
| Choline (mg) | 14 | 21 |
| Inositol (mg) | 3.9 | 4.4 |
| Carnitine (mg) | 1.7 | 1.6 |
| *Test formula: partially hydrolyzed whey infant formula; control formula: intact protein formula; DHA: Docosahexaenoic acid; GOS: galacto-oligosaccharides.* | | |
