# Supplementary material for: A Partially Hydrolyzed Whey Infant Formula Supports Appropriate Growth: A Randomized Controlled Non-Inferiority Trial
Source: Nutrients. 2020 Oct 6;12(10):3056. doi: 10.3390/nu12103056 (PMC7650565; doi:10.3390/nu12103056)
Supplement: Supplementary file 1 [file nutrients-12-03056-s001.zip › Methods, Supplementary File 3_revised.docx]

eMethods 3. Primary and secondary outcome measures and statistical analysis.

| The primary outcome was weight gain (g/day) calculated as the difference in infant weight between the baseline and the 3^rd^ follow-up visit, divided by the number of days between these visits. Secondary outcomes included other anthropometric indices assessed at each follow-up visit: weight (g), length (cm), head circumference (cm), BMI (kg/m^2^) and their Z-scores [based on the World Health Organisation (WHO) child growth standards] [^[[1]](#footnote-1)^]. Furthermore, recumbent length gain (cm/day) and head circumference gain (cm/day) from baseline to each one of the follow-up visits were calculated and compared between the two groups, while weight gain (g/day) between the baseline and the 1^st^ and 2^nd^ follow-up visits was also evaluated. Infants’ anthropometrics were measured in triplicates at baseline and at every monthly follow-up visit by the paediatricians, following standardized procedures. Specifically, the weight of each infant was recorded by weighing it three times wearing nothing but a clean diaper, on calibrated electronic scales, and if the pair-wise difference of the three measurements was more than 100 g, an additional measurement was performed. Recumbent length was measured three times using a standard measuring board, and in case of a deviation of more than 0.7 cm an additional measurement was performed. Non-stretchable slotted insertion tape was used to measure head circumference and if the pair-wise difference of the three measurements was more than 0.5 cm, an additional measurement was performed.  For the demographic, perinatal and baseline characteristics of study participants, P-values for the between groups difference (test minus control) for age at baseline were calculated using Wilcoxon Rank Sum Test; P-values for the between groups difference (test minus control) for all anthropometric indices at baseline were calculated using two Independent Sample t Tests; P-values for the between groups difference (test minus control) for all other characteristics were calculated using Cochran-Mantel-Haenszel test. The primary outcome was analyzed using an Analysis of Covariance model with study formula as fixed factor, adjusting for baseline weight, sex, antibiotics use, illnesses independent of the formula (related/non-related adverse events), smoking in the home environment, birth weight, maternal pre-pregnancy BMI, father’s current BMI, existence of gestational diabetes and if it was untreated, formula intake. The secondary outcomes were analyzed using Mixed Models Repeated Measures analysis with study formula and visit as fixed factors and adjusting for baseline anthropometry, sex, smoking in the home environment, antibiotics use, illnesses independent of the formula, birth weight, maternal pre-pregnancy BMI, father’s current BMI, existence of gestational diabetes and whether it was untreated, formula intake and significant interactions of these covariates with visit and/or study formula, with Kenward-Roger degrees of freedom and autoregressive covariance structure. For comparison of weekly formula consumption between the two groups, a Wilcoxon Rank Sum Test was used, while for analysis of the daily formula intake by body weight (mL/g/d) between the two groups an Analysis of Variance model with study formula as fixed factor was used. |
| --- |

1. WHO Multicentre Growth Reference Study Group. *WHO Child Growth Standards : Length/Height-for-Age, Weight-for-Age, Weight-for-Length, Weight-Forheight and Body Mass Index-for-Age : Methods and Development.;* 2006. doi:10.4067/S0370-41062009000400012 [↑](#footnote-ref-1)
