# Supplementary material for: A Partially Hydrolyzed Whey Infant Formula Supports Appropriate Growth: A Randomized Controlled Non-Inferiority Trial
Source: Nutrients. 2020 Oct 6;12(10):3056. doi: 10.3390/nu12103056 (PMC7650565; doi:10.3390/nu12103056)
Supplement: Supplementary file 1 [file nutrients-12-03056-s001.zip › Table, Supplementary File 4_new.docx]

eTable 4. Weight, length, head circumference and BMI at each follow-up visit by study group in the PP population

| **Study Visit** | **Test**  **(n=72)** | **Control**  **(n=70)** | **Difference of means^3^** | | | |  |
| --- | --- | --- | --- | --- | --- | --- | --- |
|  | **LS Mean (SE)** | **LS Mean (SE)** | **Difference** | **95% CI** | | **P-value^1^** | **P-value^2^** |
| **Weight, g** | | | | | | |  |
| Follow-up 1 | 6224 (158) | 6221 (149) | 2.74 | -138.23, 143.71 | 0.969 | | 0.330 |
| Follow-up 2 | 6902 (158) | 6997 (149) | -95.02 | -236.04, 46.01 | 0.185 | |  |
| Follow-up 3 | 7456 (158) | 7560 (149) | -104.05 | -244.99, 36.89 | 0.147 | |  |
| **Length, cm** | | | | | | |  |
| Follow-up 1 | 64.10 (0.57) | 63.64 (0.54) | 0.46 | -0.07, 0.99 | | 0.090 | 0.283 |
| Follow-up 2 | 66.69 (0.57) | 66.51 (0.55) | 0.18 | -0.35, 0.71 | | 0.499 |  |
| Follow-up 3 | 69.21 (0.57) | 69.08 (0.55) | 0.13 | -0.40, 0.66 | | 0.628 |  |
| **BMI, kg/m^2^** | | | | | | |  |
| Follow-up 1 | 40.15 (0.27) | 40.27 (0.25) | -0.13 | -0.37, 0.12 | | 0.307 | 0.244 |
| Follow-up 2 | 41.31 (0.27) | 41.46 (0.25) | -0.15 | -0.39, 0.10 | | 0.238 |  |
| Follow-up 3 | 42.27 (0.27) | 42.39 (0.25) | -0.12 | -0.36, 0.13 | | 0.349 |  |
| **Head circumference, cm** | | | | | | |  |
| Follow-up 1 | 15.05 (0.43) | 15.43 (0.41) | -0.38 | -0.77, 0.01 | | 0.055 | 0.064 |
| Follow-up 2 | 15.48 (0.43) | 15.85 (0.41) | -0.37 | -0.76, 0.02 | | 0.065 |  |
| Follow-up 3 | 15.55 (0.43) | 15.82 (0.41) | -0.27 | -0.66, 0.12 | | 0.171 |  |
| *^1^ between groups difference per time point*  *^2^ average treatment effect over time*  *^3^ difference in LS means between test and control formula*  *Test: partially hydrolyzed whey infant formula; control: intact protein formula; PP: per protocol; CI: confidence interval; LS mean: least squares mean; SE: standard error; BMI: body mass index.* | | | | | | | |
