# Supplementary material for: A Partially Hydrolyzed Whey Infant Formula Supports Appropriate Growth: A Randomized Controlled Non-Inferiority Trial
Source: Nutrients. 2020 Oct 6;12(10):3056. doi: 10.3390/nu12103056 (PMC7650565; doi:10.3390/nu12103056)
Supplement: Supplementary file 1 [file nutrients-12-03056-s001.zip › Table, Supplementary File 5_new.docx]

eTable 5. Weight, length, head circumference and BMI at each follow-up visit by study group in the ITT population.

| **Study Visit** | **Test**  **(n=83)** | **Control**  **(n=80)** | **Difference of means^3^** | | |  |
| --- | --- | --- | --- | --- | --- | --- |
|  | **LS Mean (SE)** | **LS Mean (SE)** | **Difference** | **95% CI** | **P-value^1^** | **P-value^2^** |
| **Weight, g** | | | | | |  |
| Follow-up 1 | 6217 (157) | 6217 (148) | 0.44 | -136.41, 137.28 | 0.995 | 0.278 |
| Follow-up 2 | 6891 (157) | 6989 (148) | -98.76 | -236.38, 38.87 | 0.158 |  |
| Follow-up 3 | 7450 (157) | 7564 (148) | -114.25 | -252.55, 24.04 | 0.105 |  |
| **Length, cm** | | | | | |  |
| Follow-up 1 | 63.99 (0.57) | 63.60 (0.54) | 0.39 | -0.13, 0.91 | 0.138 | 0.459 |
| Follow-up 2 | 66.57 (0.57) | 66.47 (0.54) | 0.10 | -0.42, 0.62 | 0.703 |  |
| Follow-up 3 | 69.10 (0.57) | 69.08 (0.54) | 0.03 | -0.50, 0.55 | 0.918 |  |
| **BMI, kg/m^2^** | | | | | |  |
| Follow-up 1 | 15.09 (0.43) | 15.43 (0.41) | -0.34 | -0.72, 0.03 | 0.075 | 0.086 |
| Follow-up 2 | 15.51 (0.43) | 15.84 (0.41) | -0.34 | -0.72, 0.04 | 0.083 |  |
| Follow-up 3 | 15.58 (0.43) | 15.82 (0.41) | -0.24 | -0.62, 0.14 | 0.214 |  |
| **Head circumference, cm** | | | | | |  |
| Follow-up 1 | 40.10 (0.27) | 40.26 (0.25) | -0.16 | -0.40, 0.08 | 0.187 | 0.144 |
| Follow-up 2 | 41.26 (0.27) | 41.43 (0.25) | -0.17 | -0.41, 0.07 | 0.153 |  |
| Follow-up 3 | 42.23 (0.27) | 42.37 (0.25) | -0.14 | -0.38, 0.10 | 0.239 |  |
| *^1^ between groups difference per time point*  *^2^ average treatment effect over time*  *^3^ difference in LS means between test and control formula*  *Test: partially hydrolysed whey infant formula; control: intact protein formula; ITT: intention to treat; CI: confidence interval; LS mean: least squares mean; SE: standard error; BMI: body mass index.* | | | | | | |
