# Supplementary material for: A Partially Hydrolyzed Whey Infant Formula Supports Appropriate Growth: A Randomized Controlled Non-Inferiority Trial
Source: Nutrients. 2020 Oct 6;12(10):3056. doi: 10.3390/nu12103056 (PMC7650565; doi:10.3390/nu12103056)
Supplement: Supplementary file 1 [file nutrients-12-03056-s001.zip › Table, Supplementary File 6_new.docx]

eTable 6. Gains in weight, length and head circumference at each follow-up visit by study group.

|  | **Population** | **Group** | **LS mean (SE)** | **Difference between groups**  **(Test vs. Control)** | | **P-value** |
| --- | --- | --- | --- | --- | --- | --- |
|  |  |  |  | Estimate | 95% CI |  |
| **Weight gain (g/d)**  Baseline – 1^st^ follow-up | PP | Test | 26.28 (2.78) | -1.71 | -4.24, 0.82 | 0.184 |
|  |  | Control | 27.99 (2.63) |  |  |  |
|  | ITT | Test | 26.17 (2.84) | -1.60 | -4.11, 0.90 | 0.207 |
|  |  | Control | 27.78 (2.68) |  |  |  |
| **Weight gain (g/d)**  Baseline – 2^nd^ follow-up | PP | Test | 24.60 (2.78) | -1.56 | -4.10, 0.97 | 0.225 |
|  |  | Control | 26.16 (2.63) |  |  |  |
|  | ITT | Test | 24.38 (2.84) | -1.63 | -4.15, 0.89 | 0.203 |
|  |  | Control | 26.01 (2.68) |  |  |  |
| **Length gain (cm/d)**  Baseline – 1^st^ follow-up | PP | Test | 0.137 (0.012) | 0.01 | -0.00, 0.02 | 0.052 |
|  |  | Control | 0.127 (0.011) |  |  |  |
|  | ITT | Test | 0.135 (0.012) | 0.01 | -0.00, 0.02 | 0.104 |
|  |  | Control | 0.126 (0.011) |  |  |  |
| **Length gain (cm/d)**  Baseline – 2^nd^ follow-up | PP | Test | 0.127 (0.012) | 0.00 | -0.01, 0.01 | 0.518 |
|  |  | Control | 0.124 (0.011) |  |  |  |
|  | ITT | Test | 0.125 (0.012) | 0.00 | -0.01, 0.01 | 0.820 |
|  |  | Control | 0.124 (0.011) |  |  |  |
| **Length gain (cm/d)**  Baseline – 3^rd^ follow-up | PP | Test | 0.124 (0.012) | 0.00 | -0.01, 0.01 | 0.494 |
|  |  | Control | 0.120 (0.011) |  |  |  |
|  | ITT | Test | 0.122 (0.012) | 0.00 | -0.01, 0.01 | 0.694 |
|  |  | Control | 0.120 (0.011) |  |  |  |
| **HC gain (cm/d)**  Baseline – 1^st^ follow-up | PP | Test | 0.041 (0.006) | -0.01 | -0.01, -0.00 | **0.040** |
|  |  | Control | 0.047 (0.005) |  |  |  |
|  | ITT | Test | 0.040 (0.006) | -0.01 | -0.01, -0.00 | 0.017 |
|  |  | Control | 0.046 (0.005) |  |  |  |
| **HC gain (cm/d)**  Baseline – 2^nd^ follow-up | PP | Test | 0.038 (0.006) | -0.00 | -0.01, 0.00 | 0.375 |
|  |  | Control | 0.040 (0.005) |  |  |  |
|  | ITT | Test | 0.037 (0.006) | -0.00 | -0.01, 0.00 | 0.241 |
|  |  | Control | 0.040 (0.005) |  |  |  |
| **HC gain (cm/d)**  Baseline – 3^rd^ follow-up | PP | Test | 0.035 (0.006) | -0.00 | -0.01, 0.00 | 0.843 |
|  |  | Control | 0.035 (0.005) |  |  |  |
|  | ITT | Test | 0.034 (0.006) | -0.00 | -0.01, 0.00 | 0.681 |
|  |  | Control | 0.035 (0.005) |  |  |  |
| *Figures in bold indicate statistically significant P-values.*  *Test: partially hydrolysed whey infant formula; control: intact protein formula; PP: per protocol; ITT: intention to treat; CI: confidence interval; LS mean: least squares mean; SE: standard error.* | | | | | | |
