# Supplementary material for: A Partially Hydrolyzed Whey Infant Formula Supports Appropriate Growth: A Randomized Controlled Non-Inferiority Trial
Source: Nutrients. 2020 Oct 6;12(10):3056. doi: 10.3390/nu12103056 (PMC7650565; doi:10.3390/nu12103056)
Supplement: Supplementary file 1 [file nutrients-12-03056-s001.zip › Figure, Supplementary File 7_new.docx]

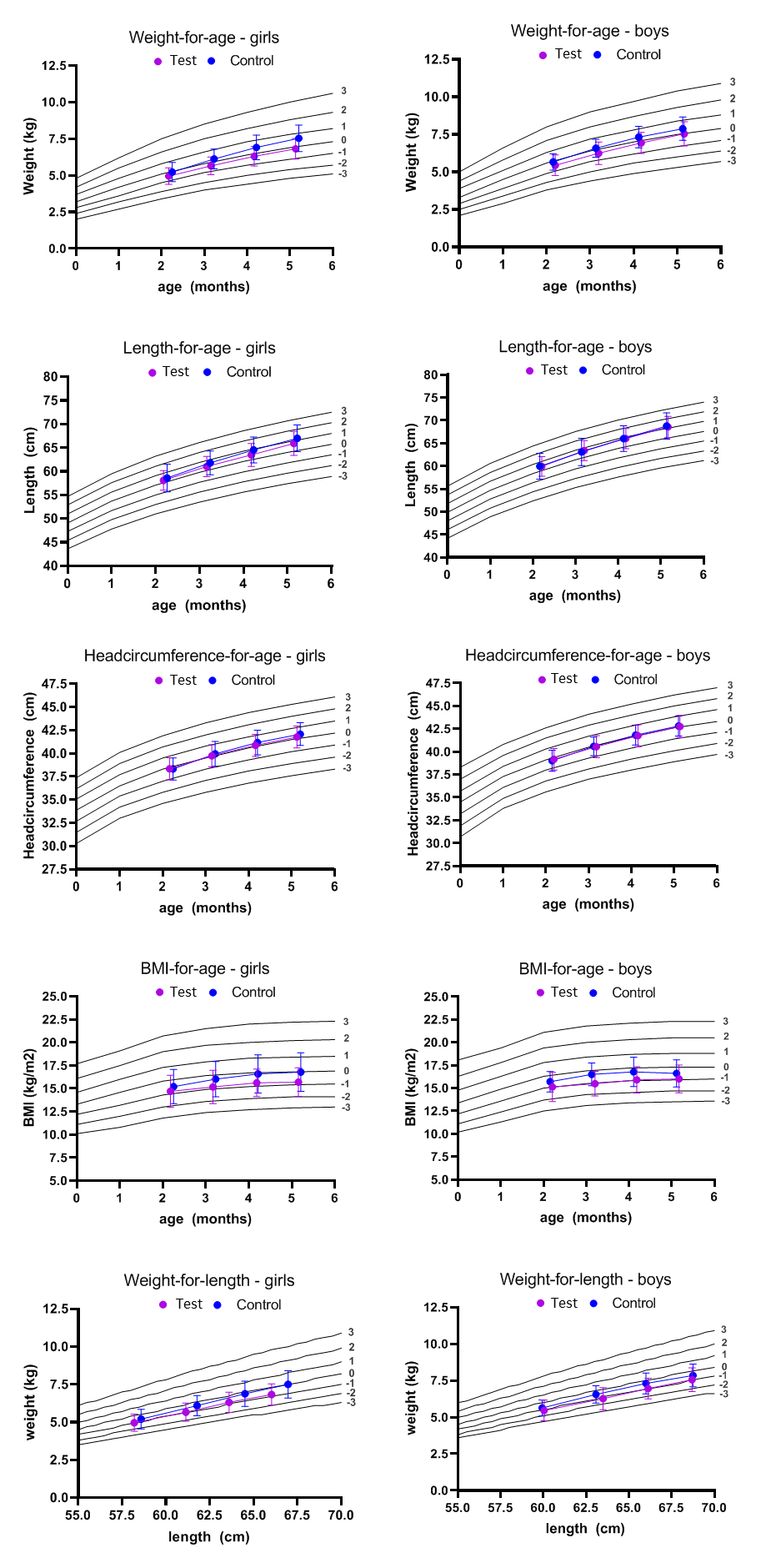


*eFigure 7. Anthropometric measurements expressed as Z-scores for weight-for-age, length-for-age, head circumference-for-age, weight-for-length and BMI-for-age during the study period in comparison with the World Health Organization growth standards for female and male infants. Test: partially hydrolyzed whey infant formula; control: standard intact protein formula.*
