# Supplementary material for: A Partially Hydrolyzed Whey Infant Formula Supports Appropriate Growth: A Randomized Controlled Non-Inferiority Trial
Source: Nutrients. 2020 Oct 6;12(10):3056. doi: 10.3390/nu12103056 (PMC7650565; doi:10.3390/nu12103056)
Supplement: Supplementary file 1 [file nutrients-12-03056-s001.zip › Table, Supplementary File 8_new.docx]

eTable 8. Overview of adverse events and serious adverse events that occurred during the trial.

|  |  | **Safety population*** | | |
| --- | --- | --- | --- | --- |
|  |  | **Group** | | |
| **Category** | **Statistic** | **Test (N=74)** | **Control (N=74)** | **Total (N=148)** |
| Adverse Events | k | 8 | 8 | 16 |
|  | n (%) | 7 (9.5) | 7 (9.5) | 14 (9.5) |
| Related Adverse Events | k | 0 | 0 | 0 |
|  | n (%) | 0 (0.0) | 0 (0.0) | 0 (0.0) |
| Discontinued Adverse Events | k | 0 | 0 | 0 |
|  | n (%) | 0 (0.0) | 0 (0.0) | 0 (0.0) |
| Serious Adverse Events | k | 5 | 2 | 7 |
|  | n (%) | 4 (5.4) | 1 (1.4) | 5 (3.4) |
| Related Serious Adverse Events | k | 0 | 0 | 0 |
|  | n (%) | 0 (0.0) | 0 (0.0) | 0 (0.0) |
| Discontinued Serious Adverse Events | k | 0 | 0 | 0 |
|  | n (%) | 0 (0.0) | 0 (0.0) | 0 (0.0) |
| *Test: partially hydrolysed whey infant formula; control: intact protein formula; N: number of subjects in analysis population; n: number of subjects with at least one event; %: percentage of subjects with at least one event, k: number of events.*  **Safety population: all infants of the ITT data analysis set minus the ones who did not consume any formula at all.* | | | | |
